# The German Arthroscopy Registry DART: what has happened after 5 years?

**DOI:** 10.1007/s00167-022-07152-7

**Published:** 2022-09-25

**Authors:** Maximilian Hinz, Christoph Lutter, Ralf Mueller-Rath, Philipp Niemeyer, Oliver Miltner, Thomas Tischer

**Affiliations:** 1 Department of Sports Orthopaedics, Technical University of Munich, Ismaninger Street 22, 81675 Munich, Germany; 2 Department of Orthopaedics, Rostock University Medical Center, Rostock, Germany; 3 OPND Orthopädische Praxisklinik, Neuss and Düsseldorf, Germany; 4 OCM Clinic, Munich, Germany; 5 DocOrtho, Berlin, Germany; 6 Department of Orthopaedic and Traumatologic Surgery, Waldkrankenhaus, Erlangen, Germany

**Keywords:** Arthroscopy, Registry, PROM, Germany, Knee, Shoulder, Hip, Ankle

## Abstract

**Purpose:**

The German Arthroscopy Registry (DART) has been initiated in 2017 with the aim to collect real-life data of patients undergoing knee, shoulder, hip or ankle surgery. The purpose of this study was to present an overview of the current status and the collected data thus far.

**Methods:**

Data entered between 11/2017 and 01/2022 were analyzed. The number of cases (each case is defined as a single operation with or without concomitant procedures) entered for each joint, follow-up rates and trends between different age groups (18–29 years, 30–44 years, 45–64 years,  ≥ 65 years) and across genders, and quality of life improvement (pre- vs. 1 year postoperative EQ visual analogue scale [EQ-VAS]) for frequently performed procedures (medial meniscus repair [MMR] vs. rotator cuff repair [RCR] vs. microfracturing of the talus [MFX-T]) were investigated.

**Results:**

Overall, 6651 cases were entered into DART, forming three distinct modules classified by joint (5370 knee, 1053 shoulder and 228 ankle cases). The most commonly entered procedures were: knee: partial medial meniscectomy (n = 2089), chondroplasty (n = 1389), anterior cruicate ligament reconstruction with hamstring autograft (n = 880); shoulder: sub acromial decompression (n = 631), bursectomy (n = 385), RCR (n = 359); ankle: partial synovectomy (n = 117), tibial osteophyte resection (n = 72), loose body removal (n = 48). In the knee and shoulder modules, middle-aged patients were the predominant age group, whereas in the ankle module, the youngest age group was the most frequent one. The two oldest age groups had the highest 1-year follow-up rates across all modules. In the knee and shoulder module, 1-year follow-up rates were higher in female patients, whereas follow-up rates were higher in male patients in the ankle module. From pre- to 1-year postoperative, MFX-T (EQ-VAS: 50.0 [25–75% interquartile range: 31.8–71.5] to 75.0 [54.3–84.3]; ∆ + 25.0) led to a comparably larger improvement in quality of life than did MMR (EQ-VAS: 70.0 [50.0–80.0] to 85.0 [70.0–94.0]; ∆ + 15.0) or RCR (EQ-VAS: 67.0 [50.0–80.0] to 85.0 [70.0–95.0]; ∆ + 18.0).

**Conclusion:**

DART has been sufficiently established and collects high-quality patient-related data with satisfactory follow-up allowing for a comprehensive analysis of the collected data. The current focus lies on improving patient enrolment and follow-up rates as well as initiating the hip module.

## Introduction

Observational studies—derived from orthopaedic registries—have helped to improve the understanding and treatment modalities of various joint pathologies, such as cartilage defects [6, 7, 16], anterior cruciate ligament tears [2, 22] and osteoarthritis [12], by analyzing large patient cohorts. Due to the fact that registries include vast, heterogeneous groups and occasionally long-term follow-up, they display real-world clinical circumstances [5, 17]. Therefore, they may be better suited to assess population health than what is considered the gold standard of research—randomized-controlled trials [20]. Randomized-controlled trials, compared to registries, usually have clear inclusion criteria, focus on rigid interventions and observe a short-term follow up. Consequently, they may be the best tool in identifying individual-oriented interventions [20]. Observational studies, on the other hand, can identify, measure and account for confounding factors, and ultimately, promote an accurate assessment of cause-effect relationships [4]. Thus, observational studies are considered as equally important as randomized-controlled trials [1, 4, 20]. In this regard, existing efforts to utilize registry data to perform orthopaedic registry-based RCTs is ongoing. This concept is especially helpful in effectiveness trials which aim to answer research questions in real-world settings [8, 11].

In an effort to prospectively assess the outcome of patients undergoing knee, shoulder, hip and ankle surgery, and ultimately, improve patient care in Germany, Austria and Switzerland, the German Arthroscopy Registry DART (www.arthroskopieregister.de) was initiated on the 15th of November 2017.

DART was introduced with multiple objectives in mind [17]. First, to collect and investigate the outcome of patients undergoing procedures under real-world clinical circumstances. Second, to identify disease- and patient-specific risk factors through subgroup analyses in order to improve patient safety. Third, to identify the impact that concomitant pathologies (commonly excluded from randomized-controlled trials) and consequently, concomitant surgical procedures have on the expected outcome (e.g., identifying the impact of meniscus surgery when performed together with anterior cruciate ligament reconstruction). This results from the fact that for various pathologies, scientific evidence, specifically outcome data, is only available for the isolated treatment and not for the combined procedure. Fourth, to compare the outcome across different pathologies and joints, e.g., microfracturing of the talus vs. medial meniscus repair vs. rotator cuff repair, using common patient-reported outcome measures (European Quality of Life 5 Dimensions 3 Level Version [EQ-5D-3L]) to report the differences in improvement stated by patients and understand which procedures lead to the highest gain for the patient.

The aim of this article is to present an overview of the collected data thus far, the progress made towards the aforementioned objectives, and the current challenges as well as future endeavours of the DART project after nearly 5 years.

## Materials and methods

### Participation in DART

The DART project was initiated by the German Society for Arthroscopy and Joint-Surgery (AGA), German Society for Arthroscopy (BVASK) and the Society for Orthopaedic Traumatologic Sports Medicine (GOTS) in cooperation with the German Knee Society (DKG) and the German Society for Orthopaedics and Trauma (DGOU) with the aim to comprehensively compile surgery- and outcome-specific data of arthroscopic knee, shoulder, hip and ankle surgeries performed in Germany, Austria and Switzerland to improve patient care. Its technical setup, structure and methodology has been previously described by Mueller-Rath et al. [17]. In brief, DART is a web-based remote data entry (RDE) system in which the surgeon and patient each complete a survey for a single case (each case is defined as a single operation with or without concomitant procedures). Depending on the treated joint, each case is classified under a single module (e.g., knee module, etc.). At baseline, the surgeon’s section includes mandatory information on patient- and joint-specific characteristics, previous operations (including the contralateral side), all surgical procedures performed on the injured joint (including defect-specific information) and therapy characteristics. The patient’s questionnaire consists of joint-specific, validated and standardized patient-reported outcome measures, such as KOOS (knee joint), ASES (shoulder joint) and FAOS (ankle joint) and a joint-independent quality of life assessment (EQ-5D-3L including the EQ visual analogue scale [EQ-VAS]) at 6-, 12-, 24-, 36-, 60- and 120-month follow-ups, as well as questions regarding satisfaction with the postoperative result [17].

Patients were eligible for participation if they were over 18 years old, surgically treated for a pathology of the knee, shoulder and/or ankle joint, signed a written consent and were in possession of a personal e-mail address in order to receive and respond to follow-up surveys.

The DART project is conducted in accordance with the Declaration of Helsinki and registered at germanctr.de (DRKS00012994). The registration of data was approved by the coordinating institutional review board of the University of Freiburg (No. 624/19) and by the local ethics committees of every participating institution.

### Objective

Data entered between the initiation of DART on the 15th of November 2017 and the 31st of January 2022 were analyzed. Specifically, (1) the number of cases entered for each joint, (2) the age distribution across different modules, (3) the 5 most commonly entered procedures, (4) follow-up rates and trends between different age groups and across genders, (5) and quality of life improvement (measured as pre- vs. 1-year postoperative EQ-VAS) for frequently performed procedures were investigated.

### Statistical analysis

Data were analyzed using SPSS 26.0 (IBM-SPSS, New York, USA). Categorical variables are presented in counts and corresponding percentages. Normal distribution of the collected continuous variables was assessed by the Shapiro–Wilk test and graphically confirmed. Accordingly, continuous variables are either presented as mean ± standard deviation (normally distributed) or median and (25–75%) interquartile range (non-normally distributed).

## Results

DART was started as a registry exclusively incorporating arthroscopic surgery. The knee registry was initiated in November 2017 and the shoulder and ankle registry were started in August and December 2018, respectively. Furthermore, in order to comprehensively display real-life clinical circumstances and build a foundation for future research, various modules were later added and also included open procedures, such as the option to add osteotomies, as well as arthroplasties into the existing knee module.

**Fig. 1:**
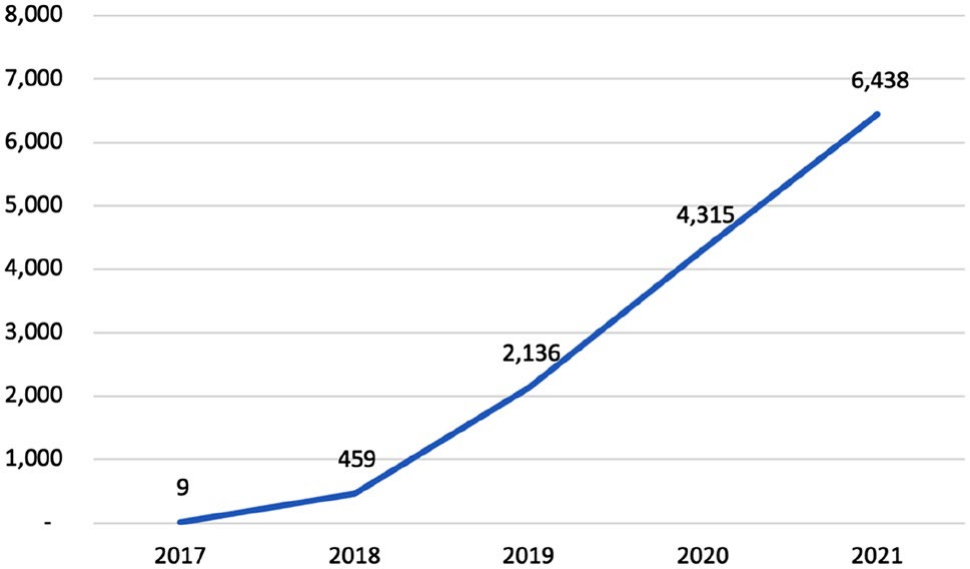
Growth in DART registry pathways between November 2017 and December 2021

**Fig. 2:**
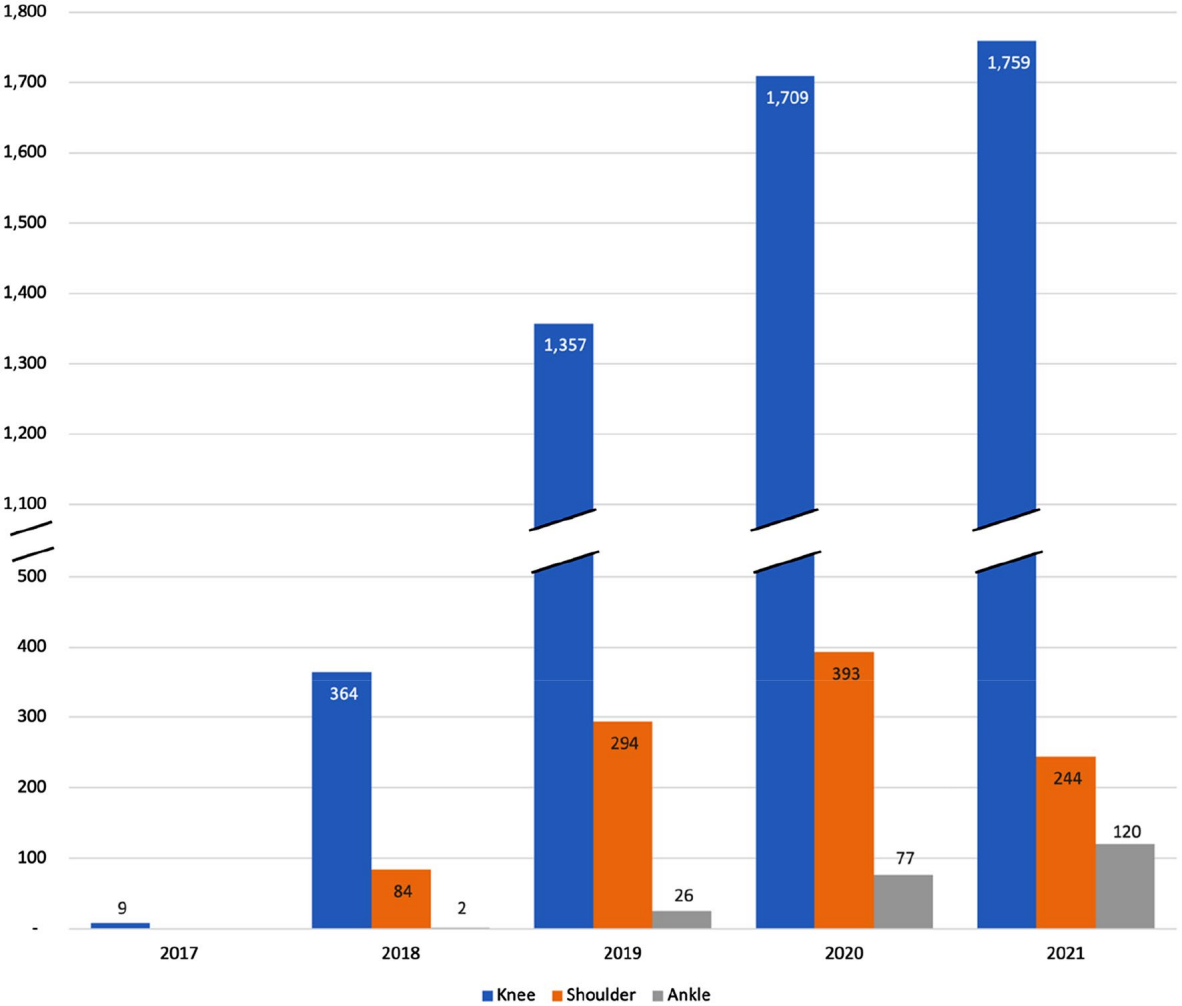
Number of pathways entered for each module per year between November 2017 and December 2021

Since the initiation of DART, a total of 6651 cases were entered as of the 31st of January 2022 (Fig. 1) with a total of 5370 knee cases, 1053 shoulder cases and 228 ankle cases (Fig. 2). Overall growth was observed every year with the exception of 2020–2021, in which the number of cases entered remained stagnant compared to the year before.

**Fig. 3:**
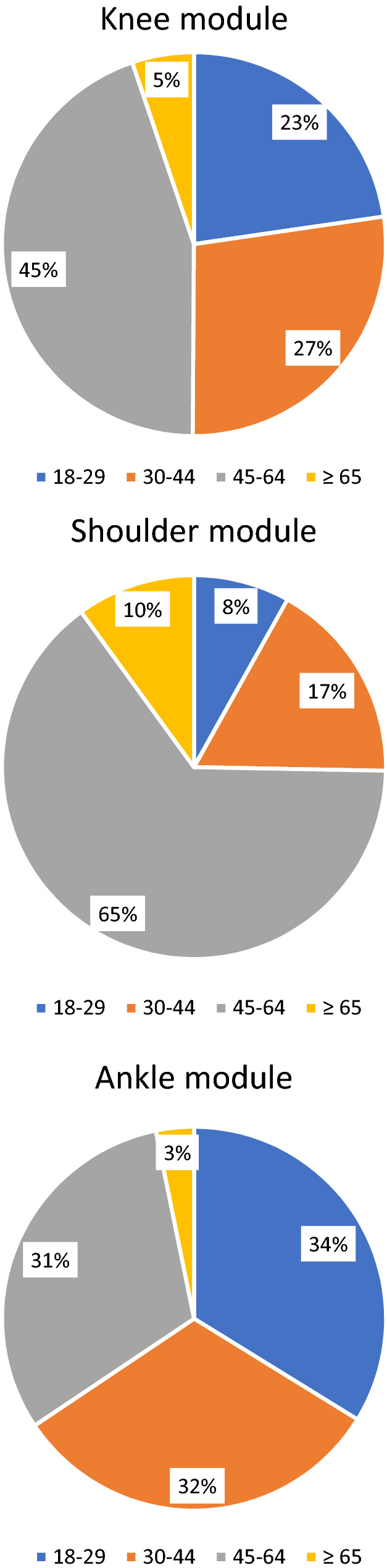
Age distribution for each module within the DART registry population. Patients of unknown age were excluded

Regarding patient demographics, the distribution of age group varied greatly between different modules (Fig. 3). Whereas the predominant age group in the knee and shoulder module were middle-aged patients (45–64 years), age groups in the ankle module were more evenly distributed—with patients aged 18–29 years being the most common age group.

An overview of the five most commonly entered procedures for each joint is presented in Table 1.

**Table 1:** List of the five most common procedures entered for each module

Procedures	*N* (in descending order)
Knee module	
Partial medial meniscectomy	2089
Chondroplasty	1389
Anterior cruciate ligament reconstruction with hamstring autograft	880
Partial synovectomy	853
Partial lateral meniscectomy	585
Shoulder module	
Subacromial decompression	631
Bursectomy	385
Rotator cuff repair	359
Long head of the biceps tendon tenotomy	156
Partial synovectomy	138
Ankle module	
Partial synovectomy	117
Tibial osteophyte resection	72
Loose body removal	48
Chondroplasty/abrasion arthroplasty of the talus	36/36
Microfracture of the talus	33

Regarding the completion of follow-up surveys, 40.1–45.7% of patients reported their outcome 6 months postoperatively, 34.5–41.7% completed the 1-year-follow-up and 32.0–36.6% participated 2 years postoperatively. There was a trend towards higher follow-up rates in older patients across all modules (Table 2). Furthermore, there was a higher 1-year follow-up rate among female participants in the knee and shoulder module (knee module: 37.9% [female participants] vs. 31.2% [male participants]; shoulder module: 69.7% [female participants] vs. 58.7% [male participants]). Only in the ankle module was the 1-year follow-up rate higher among male participants compared to female participants (54.1% [male participants] vs. 41.7% [female participants]).

**Table 2:** Age-group distribution among the knee, shoulder and ankle joint modules with 1-year follow-up rates

Age groups		*N*_respondents_/*N*_participants_	Follow-up rate (%)
Knee module			
18–29	198/449		44.1
30–44	263/504		52.2
45–64	545/907		60.1
≥ 65	64/94		68.1
Age not available	143/1567		9.1
Shoulder module			
18–29	24/39		61.5
30–44	39/75		52.0
45–64	193/304		63.5
≥ 65	39/50		78.0
Age not available	40/336		11.9
Ankle module			
18–29	7/17		41.2
30–44	8/20		40.0
45–64	15/21		71.4
≥ 65	1/1		100
Age not available	4/51		7.8

**Fig. 4:**
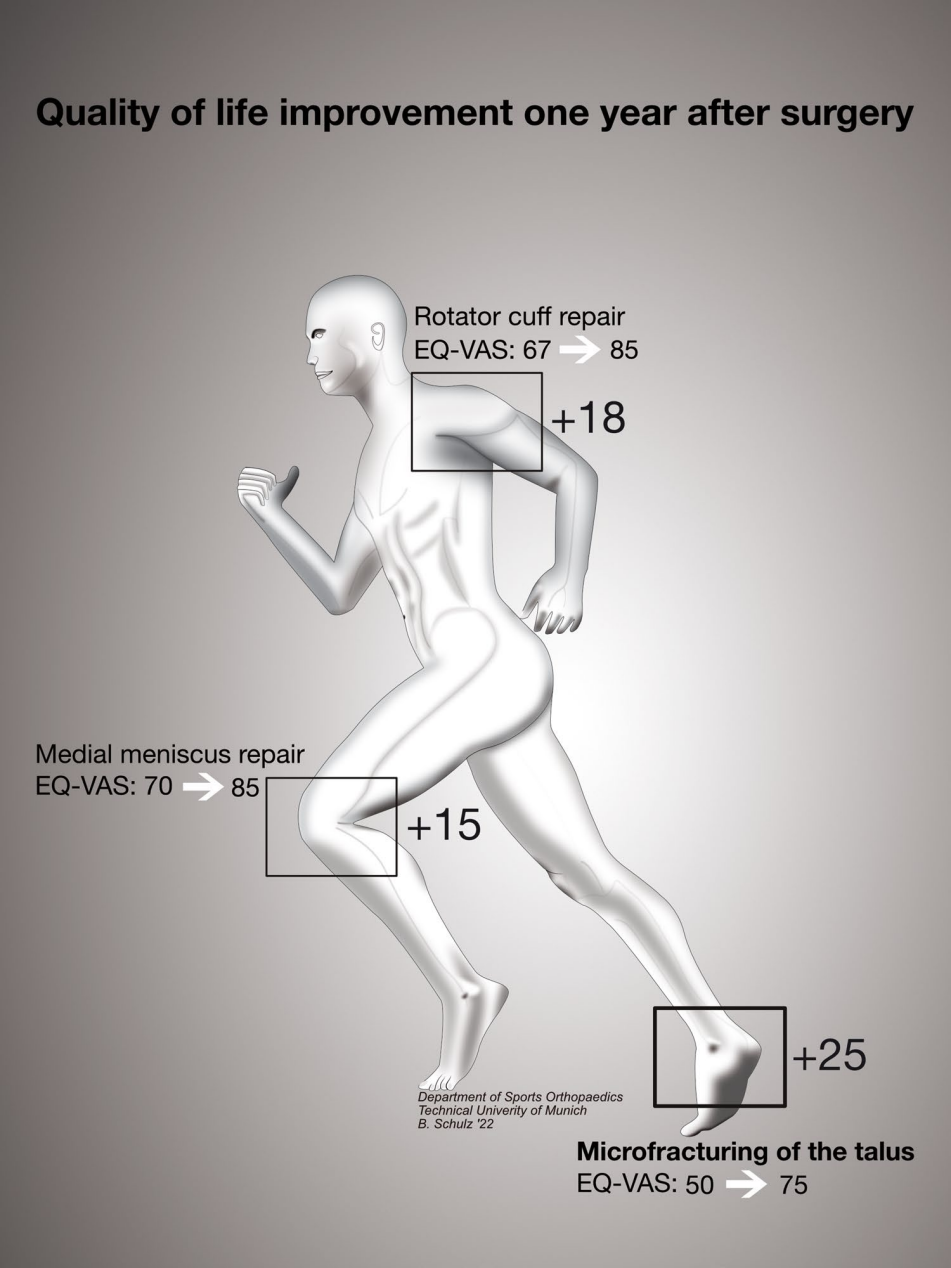
Quality of life improvement between different procedures and joints assessed using EQ-VAS

When comparing the pre- to 1-year postoperative gain in quality of life between regularly performed procedures of the knee (medial meniscus repair), shoulder (rotator cuff repair) and ankle (microfracturing of the talus), it was apparent that patients benefitted from all three procedures while microfracturing of the talus (EQ-VAS-Score: 50.0 [31.8–71.5] to 75.0 [54.3–84.3]; ∆ + 25.0) led to a comparably larger improvement in quality of life than did medial meniscus repair (EQ-VAS Score: 70.0 [50.0–80.0] to 85.0 [70.0–94.0]; ∆ + 15.0) or rotator cuff repair (EQ-VAS-Score: 67.0 [50.0–80.0] to 85.0 [70.0–95.0]; ∆ + 18.0), see Fig. 4.

## Discussion

The most important finding of this study is that DART—through its comprehensive data collection, satisfactory follow-up rates and favourable growth rate—has become an excellent database for research on patients undergoing surgery for various knee, shoulder and ankle joint pathologies within 5 years since its initiation. Although DART was initially planned as a registry exclusively collecting data on arthroscopic surgeries, the inclusion of open procedures is expected to positively influence the project’s objective of displaying real-life clinical circumstances in Germany, Austria, and Switzerland. To our knowledge, there are no other orthopaedic registries available that assess the outcome of both arthroscopic and open surgeries across different entities and joints, allowing for a direct comparison between them. Furthermore, DART enables comparison in the expected gain in life quality across surgeries in the same or different joints, which may help patients with previous orthopaedic pathologies relate between different injuries.

Nonetheless, through development, initiation and the recent data analysis of DART, several challenges became evident that needed to be addressed and will need to be addressed in the future. These challenges mainly include (1) improving DART’s acceptance and growth rate, (2) increasing data quality and follow-up rates and (3) securing funding for the DART project.

From 2017 to 2020, the annual growth rate of DART was rapidly increasing, however, between 2020 and 2021, a stagnation in growth was observed. This might be due to the negative impact of the COVID-19 pandemic on elective orthopaedic surgery as well as due to increasing work load, although the registration of each patient may only take 5–10 min [10, 13, 14]. Additionally, each institution needs approval from an institutional review board prior to patient enrolment, which may create a barrier for surgeons to start using DART. To ease this process, however, a clinical trials unit is assisting surgeons with the preparation of essential documents required for approval. Through this approach, more than 50 different institutions have participated thus far. Furthermore, to achieve a higher growth rate in 2021–2022 and into the future, significant effort is being invested in promoting DART at the annual conferences of orthopaedic societies, where its user-friendly interface and ethics committee approval support are specifically highlighted.

Beyond user growth, further efforts should be placed on improving data quality and follow-up rates. It is planned that participating providers who continuously enter cases into the DART registry will receive a certificate as well as a concise annual summary of any patient-submitted follow-up data. This may improve the clinical value of DART and consequently encourage surgeons to both include a broader spectrum of procedures into DART and also proactively and more frequently remind patients to participate.

While overall follow-up rates are comparable with the ones reported by other national arthroscopy registries (between 25 and > 75% 1 year postoperatively; [23]), there are a few points to note. First, due to the fact that the possession of an e-mail address is an inclusion criterion to participate in DART and the fact that older people are less likely to use the internet for health services [9], it was expected that older patients may be both slightly underrepresented and have lower follow-up rates than other age groups. However, across all three modules, the two oldest age groups (45–64 years and ≥ 65 years) had the highest follow-up rates. On the other hand, cases that had no information on the patient’s age had, by-far, the lowest follow-up rates across all modules (7.8–11.9% vs. 40–100%). As the patients’ age is added by the patient through the first (preoperative) survey, these patients may have never completed the preoperative survey. In an effort to improve this inconsistency, reminders for uncompleted datasets and alerts on mandatory information may be provided to both increase data quality as well as follow-up rates in the future.

Besides data quality- and volume-specific challenges, the continuation and growth of the DART project includes securing continuous funding, which, in the past and present, has been provided by orthopaedic device suppliers and orthopaedic societies. As cost-comparison studies may be initiated in the future, which may improve the economic aspects of patient care, health insurance companies may be considered to assist with the funding of the DART project.

It is estimated that around 400,000 arthroscopic procedures are performed in Germany annually. Therefore, one of DART’s limitations is the low number of cases currently represented (< 1%), indicating that the national coverage is considerably lower than for several other national arthroscopy registries (up to 97% reported, [23]). Furthermore, the number and type of procedures entered by the participating surgeons are not regulated, potentially allowing for a selection bias. It can be assumed that operations with a higher degree of difficulty are entered at a disproportionately higher level by surgeons. This issue has also been mentioned previously in the context of the German Cartilage Registry [18]. A potential countermeasure may be present in the aforementioned annual summary that participating providers would receive given that a target level of entries is reached. This would incentivize the inclusion of procedures with a lower difficulty and consequently, lower the selection bias whilst improving the coverage of procedures performed.

Another limitation of the DART project is the follow-up rate. As mentioned previously, a case is entered into DART by the surgeon immediately postoperatively for patients who have given their written consent prior to surgery. A weblink is then sent 1 day postoperatively to the participating patient’s e-mail address to collect the preoperative assessment. The issue occurs when patients do not report their preoperative assessment, and are not deleted from the DART database. These patients, as they never participated, should potentially be considered as “screening failure” instead of being categorized as “lost to follow-up”. In the future, deleting data of those patients who have not reported their preoperative assessment by a predetermined time (at which potential recollection bias may impact data quality) may be considered [26]. Nonetheless, effort should be made in order to increase the follow-up rate and decrease the rate of “screening failures”.

The DART project has several future endeavours. The fourth module, containing surgeries undertaken on the hip joint is the next important step forward and will be initiated later this year. As DART has evolved from an exclusively arthroscopic registry to a registry that also includes open procedures, the hip module will consequently also provide the option to enter hip arthroscopy and arthroplasty cases as well. Furthermore, in an effort to comprehensively analyze the success of conservative treatment modalities—primarily tendinopathies—the development of non-surgical treatment modules is currently in progress. Lastly, artificial intelligence and machine learning, as they already have been proven to be valuable tools in orthopaedic research for several different pathologies, are planned to be utilized in future DART-related research [3, 15, 19, 21, 24, 25].

## Conclusion

Within almost 5 years after its initiation, DART has been sufficiently established and collects high-quality patient-related data with satisfactory follow-up. The current focus is on, (1) improving patient enrolment and follow-up rates and (2) initiating the hip as well as (3) non-surgical treatment modules to comprehensively collect data and build the foundation for future studies.
